# Serum proteomic profiling of major depressive disorder

**DOI:** 10.1038/tp.2015.88

**Published:** 2015-07-14

**Authors:** M Bot, M K Chan, R Jansen, F Lamers, N Vogelzangs, J Steiner, F M Leweke, M Rothermundt, J Cooper, S Bahn, B W J H Penninx

**Affiliations:** 1 Department of Psychiatry, EMGO Institute for Health and Care Research and Neuroscience Campus Amsterdam, VU University Medical Centre, Amsterdam, The Netherlands; 2 Department of Chemical Engineering and Biotechnology, Institute of Biotechnology, University of Cambridge, Cambridge, UK; 3 Department of Psychiatry, University of Magdeburg, Magdeburg, Germany; 4 Department of Psychiatry and Psychotherapy, Central Institute of Mental Health, Medical Faculty Mannheim, Heidelberg University, Mannheim, Germany; 5 Department of Psychiatry, University of Münster, Münster, Germany; 6 Evangelisches Klinikum Niederrhein, Oberhausen, Germany; 7 Department of Neuroscience, Erasmus Medical Center, Rotterdam, The Netherlands

## Abstract

Much has still to be learned about the molecular mechanisms of depression. This study aims to gain insight into contributing mechanisms by identifying serum proteins related to major depressive disorder (MDD) in a large psychiatric cohort study. Our sample consisted of 1589 participants of the Netherlands Study of Depression and Anxiety, comprising 687 individuals with current MDD (cMDD), 482 individuals with remitted MDD (rMDD) and 420 controls. We studied the relationship between MDD status and the levels of 171 serum proteins detected on a multi-analyte profiling platform using adjusted linear regression models. Pooled analyses of two independent validation cohorts (totaling 78 MDD cases and 156 controls) was carried out to validate our top markers. Twenty-eight analytes differed significantly between cMDD cases and controls (*P*<0.05), whereas 10 partly overlapping markers differed significantly between rMDD cases and controls. Antidepressant medication use and comorbid anxiety status did not substantially impact on these findings. Sixteen of the cMDD-related markers had been assayed in the pooled validation cohorts, of which seven were associated with MDD. The analytes prominently associated with cMDD related to diverse cell communication and signal transduction processes (pancreatic polypeptide, macrophage migration inhibitory factor, ENRAGE, interleukin-1 receptor antagonist and tenascin-C), immune response (growth-regulated alpha protein) and protein metabolism (von Willebrand factor). Several proteins were implicated in depression. Changes were more prominent in cMDD, suggesting that molecular alterations in serum are associated with acute depression symptomatology. These findings may help to establish serum-based biomarkers of depression and could improve our understanding of its pathophysiology.

## Introduction

Major depressive disorder (MDD) is a complex, burdensome psychiatric disorder with a lifetime prevalence of about 16%.^1^ It is highly heterogeneous in terms of etiology, presentation, course and response to treatment. Several biological mechanisms have been related to MDD, including monoamine deficiency, neurotrophic alterations, dysfunctional hypothalamic–pituitary–adrenal axis activity and inflammatory alterations, but a deeper understanding of the pathophysiology of MDD is currently lacking.^2, 3^

Despite an estimated heritability of 31–42% of MDD,^4^ identification of potential genetic loci for depression appears a difficult task.^5^ Although the profiling of genes can provide a static view of potential biological pathways involved in diseases, proteins represent the functional readout in a biological system. Hence, protein profiling may better reflect the dynamic pathophysiological processes, representing both expression and post-translational modifications. Recent proteomic technologies enable simultaneous quantitative measurement of numerous proteins in individual samples. Given the complex nature of MDD, this may be of importance as one may expect the involvement of multiple rather than single markers in the pathophysiology of MDD.^6^ The application of these techniques to MDD may therefore be a powerful method to find new biomarkers of depression in an unbiased, hypothesis-free context. Furthermore, it may help to identify biological pathways involved in depression.

Current clinical proteomic research predominantly aims to identify unique protein patterns related to specific diseases, which can subsequently be used for diagnosis, prognosis or disease monitoring. For schizophrenia and bipolar disorder, this approach has resulted in the identification of specific serum protein patterns related to these disorders.^7, 8^ Few studies have simultaneously assessed such an extensive range of biomarkers in relation to the presence of MDD. Although proteomics investigations have identified changes in MDD post-mortem brain tissue and cerebrospinal fluid,^9, 10^ collecting these specimens is not feasible in routine clinical practice. Moreover, MDD not only manifests in the brain. Peripheral biological alterations have also been related to MDD. Several recent studies have investigated large numbers of peripheral molecules assessed in blood or urine, and were able to identify molecular signatures related to depression, which mainly comprised various markers involved in inflammation, insulin-related pathways and metalloproteinases.^11, 12, 13, 14, 15, 16, 17, 18^ It remains unclear, however, whether individuals with MDD in remission express similar protein profiles as individuals with current episodes, and whether altered protein levels could be ascribed to the presence of MDD or to antidepressant medication use. In addition, most existing studies were relatively small with <30 MDD patients,^16, 18^ and lacked independent validation cohorts.^11, 12, 13, 16^

We investigated whether a multi-analyte panel that quantifies serum proteins involved in hormonal, immunological and metabolic pathways may help to identify proteins and pathways associated with the presence of MDD. For this purpose, we included the largest number of participants in proteomic research on MDD so far, with 687 current MDD (cMDD) cases, 482 remitted MDD (rMDD) cases and 420 controls. Furthermore, we examined whether serum protein patterns in our sample were differentially related to various depression characteristics (that is, cMDD vs rMDD, antidepressant medication use, comorbid anxiety and depression severity), and we tested whether we could validate the association in a pooled validation cohort consisting of two independent cohorts of 78 MDD patients and 156 controls.

## Materials and methods

Data were derived from the Netherlands Study of Depression and Anxiety (NESDA), an ongoing longitudinal cohort study on the predictors, course and consequences of depressive and anxiety disorders. The NESDA sample consists of 2981 participants aged 18–65 years, comprising persons with and without depressive and/or anxiety disorders. Participants were recruited from the general population (*n*=564), primary care (*n*=1610) and specialized mental health care (*n*=807). Between September 2004 and February 2007, all participants visited one of the research centers to complete the 4-h baseline assessment, which included a face-to-face interview, written questionnaires and biological measurements. We report only the measurements used in the present paper. A detailed description of the NESDA study design can be found elsewhere.^19^

The research protocol was approved by the Ethical Committee of the participating centers, and all participants provided written informed consent.

Using the baseline assessment, we compared the serum levels of proteomic analytes of individuals with cMDD and rMDD to healthy controls. These analytes were determined in the subset of 1837 NESDA participants who participated in both baseline and 2-year follow-up assessments, and for whom sufficient serum was available (~1 ml). Of the individuals with no MDD in lifetime, we removed participants with lifetime anxiety disorder and lifetime dysthymia (236 and 3 participants, respectively) to select our healthy controls. After removing nine females with self-reported (potential) pregnancy, 1589 participants remained (687 individuals with cMDD, 482 individuals with rMDD and 420 controls).

### Major depressive disorder

During the baseline assessment, the presence of depressive disorders (MDD and dysthymia) and anxiety disorders (panic disorder, social phobia, generalized anxiety disorder and/or agoraphobia) was ascertained with the Diagnostic and Statistical Manual of Mental Disorders, Fourth Edition (DSM-IV)-based Composite Interview Diagnostic Instrument (CIDI, version 2.1, World Health Organization, 1997) by specially trained research staff. The CIDI has a high reliability and validity for the assessment of depressive and anxiety disorders.^20^ For the present study, we distinguished participants with cMDD (MDD in the past 6 months), participants with rMDD (lifetime MDD, but not in the past 6 months) and controls (no lifetime depressive and anxiety disorder according to CIDI). Comorbid anxiety disorder was defined as the presence of an anxiety disorder in the last 6 months. Severity of depression was assessed in all participants using the self-reported 30-item Inventory of Depressive Symptomatology (IDS).^21^ Furthermore, participants were asked to bring their medication containers to the visit. Antidepressant medication taken on a regular basis (at least 50% of the time) was classified using the World Health Organization Anatomical Therapeutic Chemical classification system codes as tricyclic antidepressants (N06AA), selective serotonin reuptake inhibitors (N06AB) and other antidepressants (N06AX, N06AF and N06AG).^22^

### Proteomic analytes

Blood was sampled after an overnight fast in five research centers throughout the Netherlands (Amsterdam, Leiden, Groningen, Emmen and Heerenveen), and stored at −80 °C. All samples were shipped on dry ice and processed from frozen in a Clinical Laboratory Improvement Amendments-certified laboratory (Myriad RBM; Austin, TX, USA), where a panel of 243 analytes (Myriad RBM DiscoveryMAP 250+) involved in various hormonal, immunological and metabolic pathways were assessed in serum using multiplexed microbead immunoassays (see Supplementary Table 1 for an overview of all analytes).

This method measures analytes using a flow cytometric system. The process was fully automated (for white paper http://rbm.myriad.com/scientific-literature/white-papers/quality-control-white-paper/). The analytical method has been successfully applied in various diseases.^8^ Each batch also contained three duplicate control samples with different protein concentrations, giving an average inter- and intra-assay variability of 10.6% (range 5.5–32.5%) and 5.6% (range 2.5–15.8%), respectively.

### Covariates

The following potential confounders were considered: sex, age, self-reported north-European ancestry, research center, batch number, smoking status, alcohol intake (number of drinks per week), body mass index, physical activity, corticosteroid use, anti-inflammatory medication use, sex hormone use, diabetes treatment and cardiovascular diseases treatment. Weight and height were measured by trained staff to calculate body mass index. Physical activity was assessed with the International Physical Activity Questionnaire, and expressed in 1000 metabolic equivalent minutes per week.^23^ The World Health Organization Anatomical Therapeutic Chemical coding system^22^ was used to classify regular intake (at least 50% of the time) of the following medication classes: corticosteroids (H02, R03BA, R03AK and D07), anti-inflammatory medications (M01A, M01B, A07EB and A07EC), sex hormones (G03 or self-reported use of oral contraceptives), diabetes treatment (A10 or self-reported treatment for diabetes) and cardiovascular medication (C).

### Statistical analysis

After excluding analytes with >30% missing data (mostly due to values outside the ranges of detection), 171 of the 243 analytes remained for analysis. For these 171 analytes, values that were below and above the limits of detection were imputed with the values of the lower and upper limit of detection, respectively. All analytes were log10-transformed to stabilize variance. We applied the ComBat procedure^24^ to the analytes to remove any potential batch effects. Missing values were subsequently imputed by median values (average per analyte: *n*=1).

Linear regression was used to test for an association between MDD status (control (reference), rMDD and cMDD) and each analyte (main analysis). We adjusted each model for selected potential confounders, which were derived from the stepwise procedure (forward and backward) based on Bayesian Information Criteria. Age and sex were forced to be included in each model.

In our main analyses, participants were allowed to use antidepressant medication and to have a comorbid anxiety disorder, because these are common characteristics of MDD patients and are reflective of a more severe MDD.^25^ However, the potential influence of antidepressant medication and comorbid anxiety was examined in cMDD cases by comparing analyte levels between antidepressant users and non-users, and between individuals with and without comorbid anxiety in linear regression models.

Furthermore, we conducted a series of additional analyses in which we repeated our main analysis in a subsample of individuals free of antidepressant medication to check whether our results were not driven by antidepressant medication use (additional analysis 1), in the subsample of individuals free of comorbid anxiety to check whether our results were specific for MDD and not driven by comorbid anxiety (additional analysis 2) and with depression severity IDS scores as an independent variable instead of MDD status to study the severity–response relationship (additional analysis 3). All additional analyses were adjusted for the same covariates as in the main analysis. Statistical analyses were performed using R Statistical Software (version 3.0.1).^26^ Two-sided *P*-values <0.05 were considered statistical significant. In addition, to account for multiple testing, the Benjamini and Hochberg false discovery rate (FDR) was also calculated for every protein.^27^

#### Validation

We tested whether we could validate the cMDD-associated analytes through pooled analyses (fixed-effects meta-analysis) of two independent cohorts consisting of a total of 156 healthy controls and 78 antidepressant-free cMDD patients. The validation cohorts were recruited by the University of Magdeburg in Germany. All patients fulfilled DSM-IV cMDD criteria while having no other psychiatric comorbidities. Controls were recruited from the general population and were free of psychiatric disorders. Furthermore, all participants were free of acute and chronic infections, allergies, autoimmune diseases, cancer or systemic diseases as determined by self-report, doctors' report or by physical examination. Approximately 150 serum analytes had been assayed in 2010 for biomarker identification purposes using an older version of the Human DiscoveryMAP version 2.0 (Myriad RBM), and 99 analytes passed quality control. Batch effects were removed with ComBat.^24^ Only analytes that differed significantly between cMDD and controls, or were significantly related to depression in at least two of the additional analyses, were selected for validation. The candidate analytes were tested for association with case/control status using stepwise logistic regression adjusted for covariates (age, sex and body mass index) selected based on the Bayesian Information Criteria (*P*<0.05).

#### Biological processes

The biological processes of the cMDD-associated analytes were looked up in the Human Protein Reference Database using SwissProt accession numbers.^28^ Statistical overrepresentation of a biological process was tested using PANTHER software,^29^ with the 171 tested markers as reference set and a Bonferroni correction for multiple testing.

#### Prediction model

Finally, in NESDA we investigated to what extent cMDD-related analytes improved the prediction of cMDD compared with a model with sociodemographic and lifestyle covariates only. These covariates were derived from a stepwise procedure based on Bayesian Information Criteria. Age and sex were forced to be included. We calculated area under the curve for logistic regression models predicting cMDD (reference group: controls) with and without these biomarkers using the R package PredictABEL.^30^

## Results

The sample included in the analyses consisted of 66.4% females and was on average 41.3 (s.d.=13.3) years. rMDD and cMDD subjects were slightly more often female and older than controls and, as expected, showed a less-healthy lifestyle, more antidepressant medication use and higher depression severity (Table 1).

Supplementary Table 2 shows the results of the association of MDD status with all 171 examined analytes. Linear regression showed that 28 markers were significantly different (*P*<0.05; with FDR-adjusted *q*-values of 0.09–0.30) between cMDD patients and controls, and an additional six markers differed significantly (*P*<0.05; with FDR-adjusted *q*-values of 0.26–0.72) between rMDD patients and controls. Four analytes (angiopoietin-2, insulin-like growth factor-binding protein-5 (IGFBP5), angiogenin and apolipoprotein D) overlapped.

**Figure 1:**
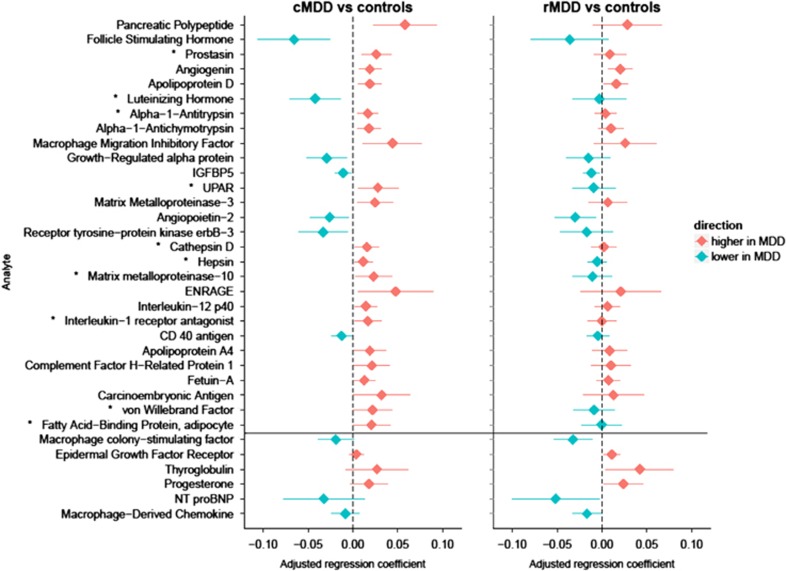
Regression coefficients and 95% confidence intervals of significant log10-transformed biomarkers in individuals with cMDD and rMDD compared with controls (reference group, total *n*=1589). Results were from linear regression analyses, all analyses were conducted separately for each log10-transformed biomarker. The figure only shows biomarkers that were statistically different in cMDD or rMDD compared with controls (*P*<0.05). Results are sorted by *P*-value. All models were adjusted for sex and age. Other relevant covariates were selected by stepwise regression. The following additional covariates were considered: ethnicity, research center, plate, smoking, alcohol intake, BMI, physical activity, corticosteroid use, anti-inflammatory drug use, sex hormone use, diabetes treatment and cardiovascular medication use. *Analytes that significantly differ between rMDD and cMDD (*P*<0.05). cMDD, current major depressive disorder; rMDD, remitted major depressive disorder.

Figure 1 shows the regression coefficients and 95% confidence intervals (CIs) for the 34 markers that were significantly different (*P*<0.05) in either cMDD patients (28 markers) or rMDD cases (10 markers), relative to controls. For 25 of the 34 analytes depicted in Figure 1, the absolute value of the regression coefficient of cMDD cases was larger than that of rMDD cases, suggesting that cMDD was more prominently related to difference in the analytes than rMDD. Of the 28 cMDD-related markers, 10 were also significantly different between cMDD and rMDD cases (prostasin, luteinizing hormone, alpha-1-antitrypsin (AAT), urokinase-type plasminogen activator receptor, cathepsin D, hepsin, matrix metalloproteinase-10, interleukin-1 receptor antagonist, von Willebrand factor and fatty acid-binding protein adipocyte (FABPA)). There were no significant differences between cMDD and rMDD patients for the six additional rMDD-related markers.

Next, we studied whether antidepressant medication use and comorbid anxiety impacted our findings. In the subgroup of cMDD subjects, only 2 of the 28 analytes differed significantly between antidepressant users vs non-users (growth-regulated alpha protein (GROa) and IGFBP5); and 2 of the 28 analytes differed significantly between cases with and without comorbid anxiety (interleukin-1 receptor antagonist and FABPA; data not shown). After excluding antidepressant medication users from our cohort, 18 analytes out of the 28 initially identified biomarkers remained related to cMDD (Table 2; additional analysis 1), suggesting that most markers are related to depression itself rather than being affected by antidepressant medication. When we excluded individuals with comorbid anxiety (Table 2; additional analysis 2), 15 analytes out of the 28 biomarkers initially identified remained significantly related to cMDD. Finally, depression severity was related to 15 out of the 28 biomarkers from the main analysis (Table 2; additional analysis 3).

Table 2 shows the results of the additional analysis for the 28 analytes that differed between cMDD and controls in the main analysis, plus the analytes that were related to depression in at least two of the additional analyses (five analytes). Of these 33 cMDD-related analytes, 25 were increased and 8 were decreased in cMDD patients compared with controls. Seven biomarkers were consistently identified in the main analysis and the three additional analyses (pancreatic polypeptide, prostasin, luteinizing hormone, AAT, macrophage migration inhibitory factor (MIF), GROa and fetuin-A). Supplementary Table 3 presents the results of the additional analyses for all 171 analytes.

### Validation

The 33 markers reported in Table 2 with *P*<0.05 were considered for validation. Of these, 16 had been assayed in the two validation cohorts (Table 3). Seven biomarkers (pancreatic polypeptide, GROa, interleukin-1 receptor antagonist, tenascin-C, von Willebrand factor, MIF and ENRAGE (extracellular newly identified receptor for advanced glycation end-products binding protein)) were significantly related to cMDD (*P*<0.05) in the same direction in NESDA as in the pooled validation cohort. The other nine proteins were either not significantly related to MDD (four analytes) or were significantly related to MDD in the opposite direction to the NESDA results (five analytes).

### Biological processes

Table 2 also shows the biological processes in which the cMDD-related analytes were involved. Most markers were involved in cell communication and signal transduction (15 markers), followed by protein metabolism (8 markers) and immune response (4 markers). The seven proteins that were validated in independent patient cohorts, covered cell communication and signal transduction (pancreatic polypeptide, MIF, ENRAGE, interleukin-1 receptor antagonist and tenascin-C), immune response (GROa) and protein metabolism (von Willebrand factor). Compared with the reference set of 171 analytes, no statistical overrepresentation of any biological process was found in PANTHER for the initially found 33 cMDD-related markers nor for the 7 validated MDD-related markers (all Bonferroni-adjusted *P*-values >0.99).

### Prediction model

Compared with a model that included sociodemographic and lifestyle covariates only (sex, age, research center, body mass index, physical activity, smoking and use of anti-inflammatory drugs), the area-under-the-curve value changed from 0.67 (95% CI 0.64–0.70) to 0.71 (95% CI 0.68–0.74) after adding the seven validated markers, and to 0.76 (95% CI 0.73-0.79) after adding all 33 cMDD-associated markers of Table 2. Supplementary Figure 1 shows the receiver-operating characteristic curves of these models.

## Discussion

We investigated whether a multi-analyte panel that quantifies serum proteins may help to identify proteins and pathways associated with MDD status. Although none of the analytes remained significantly related to cMDD after applying a strict correction for multiple testing, several proteins were found to be significantly related to MDD status using the conventional alpha-level of 0.05, with cMDD being more often related to altered proteins levels than MDD in remission. Moreover, we replicated the differences between cMDD and controls for 7 out of 16 proteins tested in an independent validation study. To the best of our knowledge, our study represents the largest proteomics study in MDD so far.

For some analytes (for example, prostasin, luteinizing hormone and AAT), associations were found with cMDD only, whereas for other analytes (angiogenin, apolipoprotein D, IGFBP5 and angiopoietin-2) associations with both cMDD and rMDD were observed. This suggests that some alterations may be depression state markers, whereas others were related to the depressive trait. The comparison of cMDD with controls has more statistical power, as the sample size of the cMDD group was larger than the rMDD group by a factor of 1.4. However, a comparison of the absolute regression coefficients of cMDD and rMDD patients, relative to controls, suggested that in general stronger associations are present for depression state than trait. A transcriptomic depression study identified nine transcripts that differentiated 32 depressed persons from 32 controls. Of these, three transcripts distinguished subjects with MDD from controls after remission following a cognitive behavioral therapy intervention.^31^ In line with our findings, these results suggest that some transcripts differentiate between MDD and controls, irrespective of the current depression status, whereas others do not.

Furthermore, despite a loss of power due to reduced sample sizes, we observed that the majority of the markers related to cMDD remained significant after exclusion of individuals who used antidepressant medication and individuals with comorbid anxiety. Within cMDD cases, both antidepressant medication use and the presence of comorbid anxiety were associated with only 2 of the 28 cMDD-related analytes (IGFPB5 and GROa for antidepressant medication; interleukin-1 receptor antagonist and FABPA for comorbid anxiety). This suggests that most of the observed associations were not driven by treatment with antidepressant medication or comorbid anxiety.

We confirmed the association for seven of the 16 candidate biomarkers that had been assayed in the validation cohorts. With respect to these seven markers, macrophage MIF, a pleiotropic cytokine, was higher in cMDD patients compared with controls. This is in line with a recent review showing that MIF was higher in persons with MDD or depressed mood, compared with non-depressed controls in five out of six studies.^32^ We further observed increased levels of interleukin-1 receptor antagonist in cMDD, which is in line with previous studies on MDD^33^ and depressive symptoms.^34^ Our observation of increased levels of von Willebrand factor, a marker involved in hemostasis, in cMDD was previously found in the study of Domenici *et al.*,^11^ and may support earlier genetic findings of an association between depressive symptoms and a genetic variant in the gene encoding the von Willebrand factor in cardiac patients.^35^

The other individual markers that we identified and replicated were not associated with MDD in previous studies, or have not been investigated. For instance, we found that pancreatic polypeptide levels were higher in cMDD patients. Pancreatic polypeptide has been linked to anorexia nervosa and conditions related to decreased food intake.^36^ Arnold *et al.*^13^ showed that another member of the pancreatic polypeptide family, peptide YY, was (marginally) positively related to depressive symptoms in older adults. In cMDD patients, we observed lower levels of the chemokine GROa. In contrast, a transcriptomic study found higher GROa levels in MDD patients in the discovery phase, but the validation cohort showed nonsignificant lower levels of GROa.^37^ Furthermore, ENRAGE was higher in cMDD patients. ENRAGE is a pro-inflammatory ligand for the receptor for advanced glycation end products, and induces inflammatory responses, migration of monocytes and macrophages and adhesion molecules.^38, 39^ Finally, tenascin-C levels were increased in cMDD. Tenascin C is increased in various cardiovascular diseases, and closely linked to tissue injury and inflammation.^40^

Although not validated in the two pooled validation cohorts, we consistently observed higher prostasin, AAT and fetuin-A levels, and lower luteinizing hormone levels in cMDD in our main analysis and three additional analyses of NESDA. Higher levels of the serine protease prostasin were previously observed in urine of MDD patients.^41^ The protease inhibitor AAT was significantly higher in MDD patients in one study^42^ but was not related to MDD in another study.^11^

Our statistical overrepresentation test indicated that no biological pathway was overrepresented among our 33 cMDD-associated markers compared with the full set of analytes under investigation. However, it is important to note that the DiscoveryMAP multi-analyte panel represents a pre-defined set of biomarkers, making statistical overrepresentation less likely. The 33 cMDD-associated markers are implicated in a wide range of biological processes, including cell communication and signal transduction, protein metabolism and immune response. Meta-analyses have linked MDD to increased levels of pro-inflammatory markers, including C-reactive protein, and interleukin-6.^43, 44^ In NESDA, previous enzyme-linked immunosorbent assay-based assessments of inflammatory markers suggested that depressed males had higher C-reactive protein levels and marginally higher interleukin-6 levels than their non-depressed counterparts.^45^ We now observed associations of cMDD with other small cytokines, such as interleukin-12p40, MIF and GROa, which is consistent with literature suggestive of inflammatory dysregulation in MDD.

The multi-analyte panel used in this study is similar to—or an updated version of—the panels employed previously to test for differences in peripheral markers between patients with other psychiatric disorders and healthy controls. In general, the results of these studies suggest more differences than overlap in markers across disorders. For example, of the 10 markers that were associated with schizophrenia in the study of van Beveren *et al.*,^46^ only one (higher levels of carcinoembryonic antigen) was related to MDD in our study. Of the 34 markers associated with schizophrenia in Schwarz *et al.*,^8^ 4 were similarly related to MDD in our study (higher levels of AAT, carcinoembryonic antigen, MIF and pancreatic polypeptide). Domenici *et al.*^11^ found that brain-derived neurotrophic factor, Rantes and epidermal growth factor were most strongly related to schizophrenia, but we found no association for these markers with MDD, and neither for the five schizophrenia markers identified in the study by Hayes *et al.*^47^ For bipolar disorder, Herberth *et al.*^7^ found 22 markers for bipolar disorder, of which 1 was correspondingly related to MDD in our sample (higher MIF). Haenisch *et al.*^48^ found 26 markers for bipolar disorder, and again only 1 was similarly associated with MDD in our study (higher cystatin C). Although these studies suggest that proteomic profiles are largely different across different psychiatric disorders, it should be noted that results were not always directly comparable because of platform or quality-control differences. Therefore, more studies are needed that directly compare the same sets of markers across psychiatric disorders.

A major advantage of our study is the large numbers of controls and MDD patients, which represent a wide variation in clinical characteristics such as depression severity, antidepressant medication use and anxiety comorbidity, reflecting the heterogeneous presentation of MDD in practice. Our findings may therefore be more generalizable to general clinical practice than studies conducted in highly selected populations. Limitations of the present study include its cross-sectional design, which impedes elucidation of the direction of the relationship between analytes and MDD. Second, analytes were only significant at relatively high FDR. However, we additionally studied the association of our cMDD-associated markers in independent cohorts to assess the external validity of our results, and were able to replicate associations for 7 out of the 16 assayed analytes, reducing the likelihood of false-positives. However, we were not able to test all eligible analytes in the validation cohorts, because not all analytes were assayed in the validation cohorts. Furthermore, no validation could be done for potential markers related to rMDD because such a validation sample was not available. Hence, future validation studies are warranted. Proteins are also known to fluctuate over time and can be influenced by many factors. We adjusted each of our analyses for the covariates that were significantly related to the protein under study, selected using stepwise regression. Although we considered a broad range of potential confounders for selection, residual confounding cannot be ruled out. Furthermore, it would be important to check whether markers are disorder specific for MDD or whether they are also altered in other psychiatric disorders, such as schizophrenia and bipolar disorder. This could not be investigated in NESDA, as these disorders were part of the exclusion criteria for participating in NESDA. Finally, conclusions can only be drawn for the selected set of molecules that were assayed by the platform.

In addition to gaining insight into MDD-related mechanisms, proteomic techniques may help to identify markers related to MDD with utility for diagnostic and prognostic purposes. Recently, an MDD diagnostic test based on nine serum markers distinguished between MDD patients and controls with a sensitivity of 91% and specificity of 81%.^42^ The replication study, involving independent MDD patients but the same controls, found nearly identical sensitivity and specificity levels.^42^ Although all nine markers were assessed in our cohort, only one marker (AAT) was significantly increased in cMDD compared with controls in NESDA, suggesting that the external validity of this diagnostic test should be further established. Further validation and refinement of our marker set could improve the clinical utility of such a diagnostic test.

Future studies are needed that link the various ‘omics'-fields to gain a better understanding of the potential pathways involved in MDD. Although the heterogeneity of MDD in our patients is consistent with the MDD patients seen in clinical practice, it may be worthwhile to additionally focus on more homogeneous MDD subtypes to identify distinct protein profiles for MDD subtypes and gain more insight in specific underlying mechanisms.

In summary, we investigated the protein profiles related to MDD in a large cohort of controls and MDD patients representing a broad range of depression severity and disease manifestations. We identified several markers predominantly involved in cell communication, signal transduction, protein metabolism and immune response that were either increased or decreased in cMDD cases compared with controls, although analytes were related to cMDD at a relatively high FDR level. Some of these markers appeared to be more strongly involved in cMDD than in rMDD, possibly representing MDD state markers. Our markers were partly replicated in independent MDD patient cohorts. Proteomic approaches may thus help to identify dysregulated pathways involved in depression.

## Figures and Tables

**Table 1:** Sample characteristics of NESDA (*n*=1589)

	*Current MDD* n=*687*		*Remitted MDD* n=*482*		*Healthy controls* n=*420*		P*-value*
Female	463	(67.4)	340	(70.5)	252	(60.0)	0.002
Age, years (mean, s.d.)	41.2	(12.3)	43.5	(12.8)	39.0	(14.9)	0.042
							
*Research center*							<0.001
Amsterdam	118	(17.2)	93	(19.3)	66	(15.7)	
Leiden	312	(45.4)	167	(34.6)	155	(36.9)	
Groningen	185	(26.9)	196	(40.7)	182	(43.3)	
Emmen	55	(8.0)	13	(2.7)	6	(1.4)	
Heerenveen	17	(2.5)	13	(2.7)	11	(2.6)	
							
Non-European ancestry	36	(5.2)	11	(2.3)	10	(2.4)	0.008
Current smoker	286	(41.6)	184	(38.2)	119	(28.3)	<0.001
Body mass index, kg/m^2^ (mean, s.d.)	26.0	(5.6)	25.9	(4.9)	24.8	(4.6)	<0.001
Alcohol intake, number of drinks per week (median, IQR)	2.4	(0.2–8.5)	3.7	(0.4–8.7)	3.7	(1.0–8.7)	<0.001
Physical activity, 1000 MET-minutes/week (median, IQR)	2.6	(1.1–4.8)	3.1	(1.5–5.7)	3.1	(1.5–5.4)	<0.001
Cardiovasular medication use	110	(16.0)	86	(17.8)	61	(14.5)	0.397
Diabetes treatment	32	(4.7)	17	(3.5)	13	(3.1)	0.376
Corticosteroid use	53	(7.7)	31	(6.4)	17	(4.0)	0.050
Anti-inflammatory drug use	38	(5.5)	25	(5.2)	4	(1.0)	<0.001
Sex hormone use	142	(20.7)	90	(18.7)	92	(21.9)	0.472
Depression severity, IDS score (median, IQR)	32	(23–40)	16	(10–25)	6	(3–12)	<0.001
							
*Antidepressant medication use*							
No	389	(56.6)	384	(79.7)	417	(99.3)	<0.001
TCA	28	(4.1)	13	(2.7)	0	(0.0)	<0.001
SSRI	203	(29.5)	73	(15.1)	3	(0.7)	<0.001
Other antidepressants^a^	77	(11.2)	15	(3.1)	0	(0.0)	<0.001
Current anxiety disorder	426	(62.0)	173	(35.9)	0	(0.0)	<0.001

Abbreviations: ANOVA, analysis of variance; IDS, Inventory of Depressive Symptomatology; IQR, interquartile range; MDD, major depressive disorder; NESDA, Netherlands Study of Depression and Anxiety; SSRI, selective serotonin reuptake inhibitor; TCA, tricyclic antidepressant.

a Other antidepressants included serotonin norepinephrine reuptake inhibitors, tetracyclic antidepressants and monoamine oxidase inhibitors.

The table shows *n* (%), unless otherwise reported. *P*-values are from a *χ*^2^-test for categorical variables, ANOVA for continuous variables and Kruskall–Wallis tests for non-normal continuous variables.

**Table 2:** Robustness of significant log10-transformed analytes related to current depression^a^

		*Main analysis*			*Additional analysis 1: exclusion of antidepressant users*			*Additional analysis 2: exclusion of individuals with comorbid anxiety*			*Additional analysis 3: depression severity*		
	*Total* n *in analyses*	n=*1589*			n=*1190*			n=*990*			n=*1577*		
		*cMDD vs control (ref)*			*cMDD vs control (ref)*			*cMDD vs control (ref)*			*10-Point increase in IDS*		
*Analyte*	*Biological process*^b^	b	P	q	b	P	q	b	P	q	b	p	q
Pancreatic polypeptide	CC,ST	0.06	**1.2** × **10**^−^**^3^**	0.09	0.04	**0.04**	0.32	0.04	**0.05**	0.32	0.015	**3.2** × **10**^−^**^3^**	0.11
FSH	CC,ST	−0.07	**1.4** × **10**^−^**^3^**	0.09	−0.06	**0.01**	0.16	−0.06	**0.02**	0.26	−0.009	0.13	0.41
Prostasin	PM	0.03	**1.9** × **10**^−^**^3^**	0.09	0.03	**2.1** × **10**^−^**^3^**	0.10	0.03	**2.3** × **10**^−^**^3^**	0.10	0.006	**0.01**	0.21
Angiogenin	M	0.02	**2.9** × **10**^−^**^3^**	0.09	0.02	**7.4** × **10**^−^**^4^**	0.10	0.02	**2.0** × **10**^−^**^3^**	0.10	0.003	0.08	0.34
Apolipoprotein D	T	0.02	**3.0** × **10**^−^**^3^**	0.09	0.02	**0.01**	0.16	0.02	**0.03**	0.28	0.003	0.09	0.36
Luteinizing hormone	CC,ST	−0.04	**3.3** × **10**^−^**^3^**	0.09	−0.04	**0.01**	0.21	−0.04	**0.04**	0.28	−0.009	**0.04**	0.25
Alpha-1-antitrypsin	PM	0.02	**3.8** × **10**^−^**^3^**	0.09	0.02	**0.02**	0.28	0.02	**0.02**	0.28	0.004	**0.01**	0.17
Alpha-1-antichymotrypsin	PM	0.02	**0.01**	0.15	0.02	**2.8** × **10**^−^**^3^**	0.10	0.03	**1.3** × **10**^−^**^3^**	0.10	0.003	0.08	0.35
Macrophage migration inhibitory factor	CC,ST	0.04	**0.01**	0.16	0.05	**4.5** × **10**^−^**^3^**	0.13	0.05	**0.02**	0.26	0.010	**0.04**	0.25
Growth-regulated alpha protein	IM	−0.03	**0.01**	0.19	−0.04	**1.6** × **10**^−^**^3^**	0.10	−0.04	**0.01**	0.26	−0.008	**0.02**	0.21
Insulin growth factor-binding protein-5	CC,ST	−0.01	**0.01**	0.19	−0.01	0.17	0.52	−0.01	**0.04**	0.28	−0.003	**0.02**	0.21
Urokinase-type plasminogen activator receptor	CC,ST	0.03	**0.01**	0.19	0.02	0.08	0.38	0.02	0.26	0.65	0.010	**3.2** × **10**^−^**^3^**	0.11
Matrix metalloproteinase-3	PM	0.02	**0.01**	0.19	0.03	**0.02**	0.26	0.02	0.19	0.61	0.006	**0.05**	0.29
Angiopoietin 2	CC,ST	−0.03	**0.02**	0.19	−0.02	0.08	0.38	−0.03	0.06	0.32	−0.006	0.07	0.33
Receptor tyrosine-protein kinase erbB-3	CC,ST	−0.03	**0.02**	0.19	−0.02	0.15	0.52	−0.05	**0.01**	0.17	−0.002	0.61	0.81
Cathepsin D	PM	0.02	**0.02**	0.22	0.01	**0.04**	0.32	0.02	0.05	0.32	0.003	0.08	0.35
Hepsin	PL	0.01	**0.02**	0.23	0.01	**0.05**	0.32	0.01	0.06	0.32	0.003	**0.03**	0.21
Matrix metalloproteinase-10	PM	0.02	**0.03**	0.23	0.02	0.06	0.37	0.02	0.24	0.64	0.005	0.11	0.39
ENRAGE	CC,ST	0.05	**0.03**	0.23	0.04	0.09	0.41	0.03	0.33	0.70	0.008	0.19	0.47
Interleukin-12p40	IM	0.01	**0.03**	0.23	0.01	**0.04**	0.32	0.01	0.41	0.77	0.004	**0.02**	0.21
Interleukin-1 receptor antagonist	CC,ST	0.02	**0.03**	0.23	0.01	0.31	0.66	0.00	0.84	0.94	0.005	**0.02**	0.21
CD40 antigen	CC,ST	−0.01	**0.03**	0.25	−0.01	0.06	0.37	−0.02	**0.01**	0.17	−0.001	0.70	0.86
Apolipoprotein A4	T	0.02	**0.04**	0.26	0.03	**0.01**	0.15	0.01	0.19	0.61	0.003	0.23	0.54
Complement factor H-related protein 1	IM	0.02	**0.04**	0.26	0.02	**0.05**	0.32	0.02	0.12	0.51	0.005	0.10	0.37
Fetuin-A	CC,ST	0.01	**0.04**	0.29	0.02	**0.01**	0.15	0.02	**0.01**	0.23	0.004	**0.02**	0.21
Carcinoembryonic antigen	IM	0.03	**0.05**	0.30	0.03	0.17	0.52	0.01	0.48	0.80	0.010	**0.04**	0.25
von Willebrand factor	PM	0.02	**0.05**	0.30	0.03	**0.03**	0.28	0.02	0.21	0.61	0.008	**0.01**	0.21
Fatty acid-binding protein, adipocyte	CC,ST	0.02	**0.05**	0.30	0.02	0.16	0.52	0.04	**3.4** × **10**^−^**^3^**	0.11	0.006	0.06	0.29
													
Cystatin C	PM	0.01	0.08	0.36	0.02	**2.4** × **10**^−^**^3^**	0.10	0.01	**0.03**	0.28	0.001	0.32	0.66
Lactoylglutathione lyase	M	0.02	0.14	0.45	0.04	**0.02**	0.21	0.04	**0.04**	0.28	0.001	0.75	0.89
Cellular fibronectin	CG	0.03	0.06	0.30	0.04	**0.03**	0.28	0.04	**0.04**	0.28	0.001	0.72	0.88
Tenascin C	CC,ST	0.02	0.10	0.38	0.02	**0.03**	0.28	0.01	0.22	0.62	0.005	**0.04**	0.25
Vascular endothelial growth factor	CC,ST	−0.02	0.07	0.35	−0.03	**0.03**	0.28	−0.03	**0.03**	0.28	−0.005	0.14	0.41

Abbreviations: b, unstandardized regression coefficient; CC, cell communication; CG, cell growth or maintenance; cMDD, current major depressive disorder; ENRAGE, extracellular newly identified receptor for advanced glycation end-products binding protein; FSH, follicle-stimulating hormone; IDS, Inventory of Depressive Symptomatology; IM, immune response; M, metabolism; PL, proteolysis and peptidolysis; PM, protein metabolism; ST, signal transduction; T, transport.

All linear regression analyses were conducted separately for each biomarker and adjusted for the same set of covariates as Figure 1 (Supplementary Table 2). Table only shows biomarkers that were significantly related to cMDD in the main analysis (28 markers) or biomarkers that were related to MDD in at least two out of the three additional analyses (five markers). All significant p-values (*P*<0.05) are in bold.

a Results for remitted MDD were omitted from the table.

b From the Human Protein Reference Database.

**Table 3:** Sample characteristics and overview of validated biomarkers in the two pooled validation cohorts

	*MDD cases*	*Controls*	
*n*	78	156	
Country	Germany	Germany	
Source of population	Psychiatric inpatient/outpatient	General population	
Depression diagnosis	MDD	None	
Males/females	27/51	76/80	
Age, mean (s.d.)	39.8 (12.8)	37.4 (11.5)	
Ethnicity	Caucasian	Caucasian	
Smoking (yes/no/NA)	36/42	39/117	
BMI, mean (s.d.)	24.7 (5.4)	25.3 (4.3)	
Antidepressant medication use	>6 Weeks off medication or drug naive first onset	None	
Other psychiatric disorders	None	None	

*Analyte*	*log(odds)*	P	*Validated?*^a^
Pancreatic polypeptide	1.60	3.03 × 10^−^^5^	Yes
FSH	1.02	0.001	No
LH	0.72	0.038	No
Alpha-1-antitrypsin	−0.06	0.96	No
Macrophage migration inhibitory factor	3.55	1.80 × 10^−^^7^	Yes
Growth-regulated alpha protein	−1.91	0.033	Yes
Matrix metalloproteinase-3	−0.58	0.29	No
Angiopoietin 2	−0.39	0.46	No
ENRAGE	2.94	4.20 × 10^−^^9^	Yes
IL12p40	−1.08	0.028	No
IL1ra	3.97	1.49 × 10^−^^6^	Yes
CD40 antigen	4.73	0.004	No
Carcinoembryonic antigen	0.61	0.066	No
von Willebrand factor	1.66	0.010	Yes
Tenascin C	2.61	0.003	Yes
Vascular endothelial growth factor	1.87	0.045	No

Abbreviations: BMI, body mass index; ENRAGE, extracellular newly identified receptor for advanced glycation end-products binding protein; FSH, follicle-stimulating hormone; LH, luteinizing hormone; MDD, major depressive disorder; NA, not applicable; NESDA, Netherlands Study of Depression and Anxiety.

a Validated: analytes were considered to be validated when the analyte was significantly related to MDD in the same direction (that is, positive or negative) as in NESDA. The following 17 markers were eligible for replication but had not been assessed in the validation cohorts: prostasin, angiogenin, apolipoprotein D, IGFBP5, urokinase-type plasminogen activator receptor, receptor tyrosine-protein kinase erbB-3, cathepsin D, fetuin-A, cystatin C, lactoylglutathione lyase, alpha-1-antichymotrypsin, hepsin, matrix metalloproteinase-10, apolipoprotein A4, complement factor H-related protein 1, fatty acid-binding protein adipocyte and cellular fibronectin.

Results from logistic regression with MDD status (0=control and 1=MDD case) as outcome.

## Notes

SB and JC have been consultants for Myriad Genetics Inc. until June 2014. SB is the director of Psynova Neurotech. JC is a consultant for Psynova Neurotech. The remaining authors declare no conflict of interest.
