# Supplementary figures and images for: Serum proteomic profiling of major depressive disorder
Source: Transl Psychiatry. 2015 Jul 14;5(7):e599–. doi: 10.1038/tp.2015.88 (PMC5068719; doi:10.1038/tp.2015.88)

ROC plot

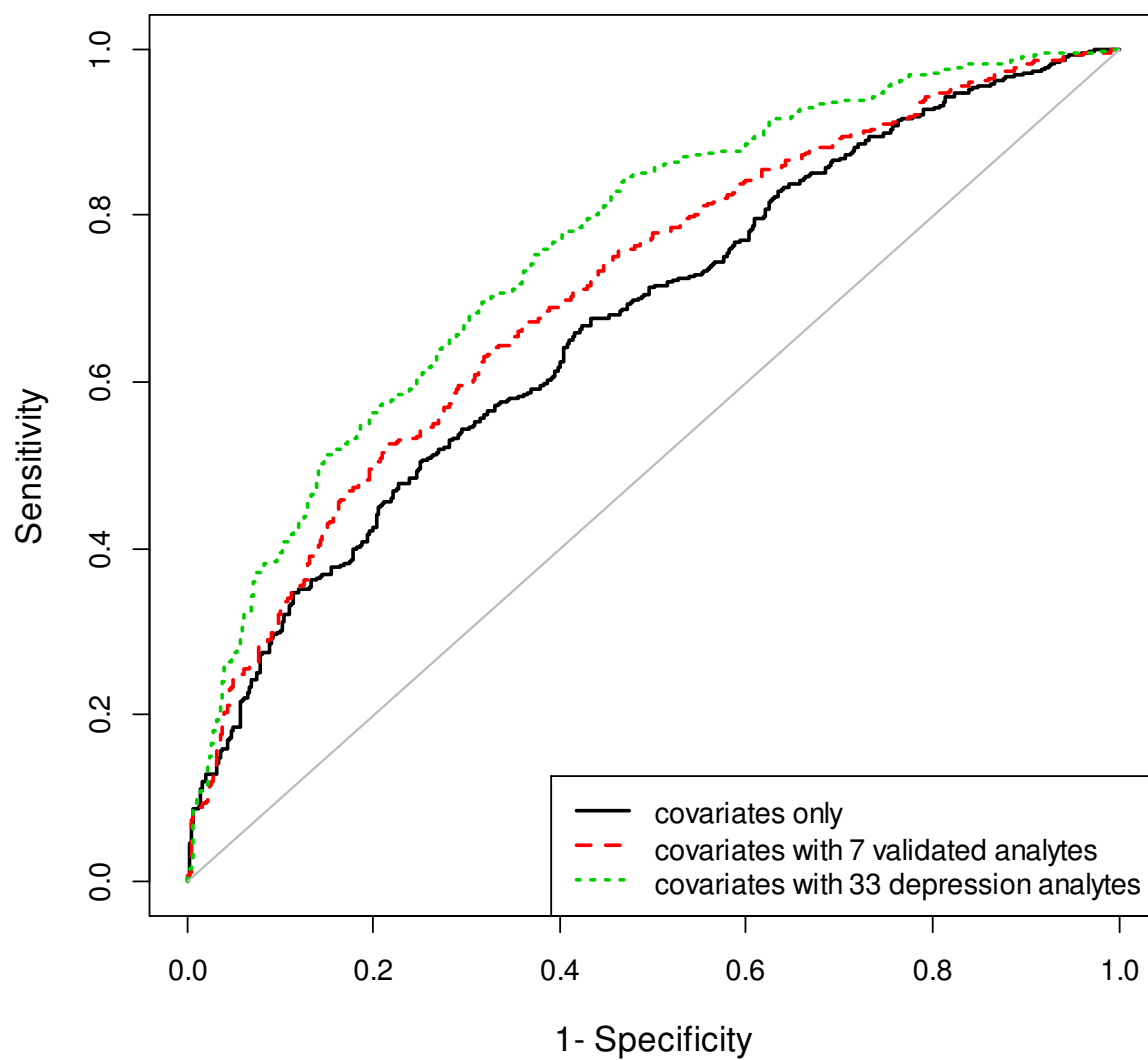

Supplement: Supplementary Figure 1 [file tp201588x1.pdf]
