# Supplementary material for: Serum proteomic profiling of major depressive disorder
Source: Transl Psychiatry. 2015 Jul 14;5(7):e599–. doi: 10.1038/tp.2015.88 (PMC5068719; doi:10.1038/tp.2015.88)
Supplement: Supplementary Table and Figure Legends [file tp201588x5.doc]

**Supplemental Table 1. Overview of all measured analytes in NESDA with Myriad RBM Discovery MAP 250+**

**Supplemental Table 2. Results of stepwise regression models for the association between MDD status (cMDD, rMDD and controls) and all 171 log10 transformed analytes**

**Supplemental Table 3. Robustness of the association between depression and all 171 log-10 transformed analytes by considering antidepressant medication use (additional analysis 1), comorbid anxiety disorder (additional analysis 2) and depression severity (additional analysis 3)**

**Supplemental Figure 1. Three receiver operating characteristic curves for distinguishing cMDD cases and controls**

The black line represents a model including significant sociodemographic and health-related variables (model 1: sex, age, research center, body mass index, physical activity, smoking and anti-inflammatory drugs), the red line represents model 1 plus the seven validated markers (model 2), and the green line represents model 1 plus the 33 depression-related markers identified in NESDA (model 3).
